# Sibling rivalry: related bacterial small RNAs and their redundant and non-redundant roles

**DOI:** 10.3389/fcimb.2014.00151

**Published:** 2014-10-28

**Authors:** Clayton C. Caswell, Amanda G. Oglesby-Sherrouse, Erin R. Murphy

**Affiliations:** ^1^Department of Biomedical Sciences and Pathobiology, Center for Molecular Medicine and Infectious Diseases, VA-MD Regional College of Veterinary Medicine, Virginia TechBlacksburg, VA, USA; ^2^Department of Pharmaceutical Sciences, School of Pharmacy, University of MarylandBaltimore, MD, USA; ^3^Department of Microbiology and Immunology, School of Medicine, University of MarylandBaltimore, MD, USA; ^4^Department of Biomedical Sciences, Ohio University Heritage College of Osteopathic MedicineAthens, OH, USA

**Keywords:** bacterial small RNA, sRNA, sibling sRNA, ribo-regulation, regulation

## Abstract

Small RNA molecules (sRNAs) are now recognized as key regulators controlling bacterial gene expression, as sRNAs provide a quick and efficient means of positively or negatively altering the expression of specific genes. To date, numerous sRNAs have been identified and characterized in a myriad of bacterial species, but more recently, a theme in bacterial sRNAs has emerged: the presence of more than one highly related sRNAs produced by a given bacterium, here termed sibling sRNAs. Sibling sRNAs are those that are highly similar at the nucleotide level, and while it might be expected that sibling sRNAs exert identical regulatory functions on the expression of target genes based on their high degree of relatedness, emerging evidence is demonstrating that this is not always the case. Indeed, there are several examples of bacterial sibling sRNAs with non-redundant regulatory functions, but there are also instances of apparent regulatory redundancy between sibling sRNAs. This review provides a comprehensive overview of the current knowledge of bacterial sibling sRNAs, and also discusses important questions about the significance and evolutionary implications of this emerging class of regulators.

## Introduction

The coordinated and timely regulation of gene expression is essential for the capacity of bacteria to sense and respond to their surroundings, particularly in highly variable and often stressful environments. As such, bacteria have evolved numerous mechanisms to control gene expression in response to specific environmental cues. One way in which bacteria control gene expression is by employing regulatory small RNAs (sRNAs), which use a variety of molecular mechanism to modulate the expression of specific gene targets (Storz et al., 2011). Many sRNA molecules control target gene expression by binding to complementary sequences within specific mRNA molecules, thereby altering the stability of, and/or translation from, the targeted mRNA. Interactions between sRNAs and their specific target(s) can result in either positive or negative effects on the expression of the regulated gene(s). In cases of positive regulation, sRNAs can bind to and alter the secondary structures of the mRNA, leading to the unmasking of a ribosome-binding site that can then be accessed by ribosomes to allow for efficient translation. Recent studies also show that sRNA-mRNA interactions can positively affect gene expression by stabilizing mRNA intermediates and full-length mRNAs (Fröhlich et al., 2013; Papenfort et al., 2013). Alternatively, inhibitory sRNAs can bind to target mRNAs resulting in destabilization and subsequent degradation of the mRNA, or the sRNA-mRNA interaction can lead to occlusion of the ribosome-binding site in the mRNA, effectively inhibiting translation of the target gene (Aiba, 2007). In addition to directly affecting mRNA stability, structure and translation, sRNAs can also control target gene expression by interacting with, and altering the function of, post-transcriptional regulatory proteins (Storz et al., 2011).

Bacteria from numerous genera and species have been shown to encode and produce sRNAs, and a large amount of literature has been published describing the regulatory and functional aspects of these important molecules (Gottesman and Storz, 2011; Bobrovskyy and Vanderpool, 2013; Lalaouna et al., 2013; Oglesby-Sherrouse and Murphy, 2013; Michaux et al., 2014). Nonetheless, while a great number of sRNAs have been identified in diverse bacteria, many questions remain regarding their function and evolution. More recently, there has been an increased recognition and characterization of examples where two or more highly related sRNAs are produced by the same bacterium. The high degree of sequence relatedness (i.e., sequence identity) between the identified sRNAs has lead to their designation here as “sibling sRNAs.” Given the significant conservation of nucleotide sequence between individual sibling sRNAs, it might be expected that these molecules perform redundant regulatory functions, such as binding to and similarly affecting the expression of identical regulons. However, while it has been shown that some sibling sRNAs do exert overlapping regulatory functions, it is clear that some sibling sRNAs perform unique, non-redundant functions within the bacterium in which they are produced. In the latter case, non-redundant functions of these sibling sRNAs may be the result of differential regulation of the sibling sRNA-encoding genes, varying mRNA targets between the siblings, and/or mechanisms of regulating gene expression that are unique to each sibling sRNA.

This review describes the current knowledge of bacterial sibling sRNAs. Specifically, we provide up-to-date overviews of specific sibling sRNA groups (Table 1). Additionally, we discuss some of the major questions surrounding bacterial sibling sRNAs, such as the evolutionary origins of these systems, as well as the biological implications of a bacterium retaining redundant and non-redundant sibling sRNAs.

**Table 1:** **Summary of bacterial sibling sRNAs characterized to date**.

**Sibling sRNA**	**Organism(s)**	**No. of Siblings**	**Genetic organization**	Redundant target(s)^*^	**Non-redundant target(s)^∧^**	**Differential regulation**	**Regulated process(es)^%^**
RyhB1 and RyhB2	*Yersinia* sp.	2	Distal	NK	NK	✓	NK
RfrA and RfrB	*S. enterica*	2	Distal	✓	✓	✓	Iron homeostasis
							Motility
							Growth within macrophages
PrrF1 and PrrF2	*P. aeruginosa*	2	Tandem	✓	✓^#^	NK	Iron homeostasis
PrrF1 and PrrF2	*Pseudomonas* sp. (non-*aeruginosa*)	2	Distal	NK	NK	NK	Iron homeostasis
OmrA and OmrB	*E. coli*	2	Tandem	✓	NK	✓	Iron acquisition
							Curli formation
							Motility
AbcR1 and AbcR2	*A. tumefaciens*	2	Tandem	NK	✓	NK	ABC transporters
AbcR1 and AbcR2	*B. abortus*	2	Distal	✓	NK	NK	ABC transporters
AbcR1 and AbcR2	*S. meliloti*	2	Tandem	NK	✓	✓	ABC transporters
Qrr	*Vibrio* sp.	4-5	Distal	✓	✓	✓	Quorum sensing
csRNA	*Streptococcus* sp.	2-6	Distal/tandem	✓	✓	NK	Autolysis
							Competence
							β-lactam resistance Virulence
GlmY and GlmZ	*E. coli*	2	Distal	NK	✓	✓	Cell wall synthesis
							Attachment
6S RNA	*B. subtilis*	2	Distal	✓	✓	✓	Sporulation
6S RNA	*L. pneumophila*	2	Distal	NK	NK	✓	Intracellular growth
							Stress response
							Virulence
Csr/Rsm	Gram-negative bacteria	2-3	Distal	✓	✓	✓	Metabolism
							Virulence

* *Redundant function indicates that the sibling sRNAs share one or more regulatory target and/or that they have been shown to influence the same bacterial process*.

∧ *Non-redundant function indicates that the sibling sRNA have one or more unique regulatory target and/or that they have been shown to influence different bacterial processes*.

% *Regulatory targets as experimentally confirmed or predicted by in silico analyses*.

# *The non-redundant nature of P. aeruginosa PrrF molecules results from the tandem arrangement and subsequent production of PrrH by this species*.

*NK: not known at the time of this review*.

## Sibling RyhB and RyhB-like sRNAs

The iron-repressed RyhB sRNA was first identified in *Escherichia coli* by Masse and colleagues in 2002 and has been shown in subsequent studies to play a critical role in regulating iron homeostasis of this model bacterium (Masse et al., 2007). This function is largely achieved through the negative regulation of genes encoding iron-containing proteins, such as iron co-factored superoxide dismutase, succinate dehydrogenase, and ferritin (Masse and Gottesman, 2002). RyhB has also been shown to mediate positive regulation of certain target genes, as is the case for *shiA*, encoding a permease of siderophore precursors (Prevost et al., 2007). As such, RyhB not only spares intracellular iron stores when iron is limiting, but also enhances the ability of the cell to acquire iron.

At the time of the first report on RyhB, the conservation of the *ryhB* sequence in many enterobacteriaceae was noted (Masse and Gottesman, 2002). Subsequent analyses have revealed that “RyhB-like” sRNAs are produced by several more distantly related bacterial species (Wilderman et al., 2004; Mellin et al., 2007; Gaballa et al., 2008; Jung and Kwon, 2008). RyhB-like sRNAs are iron-regulated small RNAs that do not share significant homology but that possess analogous regulatory function to RyhB. Together these finding indicate that sRNA-mediated iron sparing responses are conserved across a broad range of bacterial species.

Unlike *E. coli*, which encodes for a single RyhB, several bacterial species have been found to encode more than one RyhB or RyhB-like sRNA molecule. Much work has been performed in recent years to determine the roles that these sibling RyhB and RyhB-like sRNA molecules play in the physiology and, in some cases, virulence of each organism in which they are produced. In all cases examined so far, sibling RyhB homologs and analogs down-regulate the expression of iron-containing proteins when iron becomes limiting, likely sparing intracellular iron stores (Jacques et al., 2006; Oglesby-Sherrouse and Murphy, 2013). Notably, studies of several of RyhB and RyhB-like sRNAs indicate that the acquisition of a sibling allows for additional regulatory activities, with interesting implications for regulation of virulence.

### *Salmonella* Rfr

The original study of *E. coli* RyhB reported that *Salmonella enterica* possesses two genetic loci with significant homology to *E. coli ryhB*. One of the *ryhB* orthologs was identified in the same genetic context of *E. coli* RyhB, while the second ortholog was encoded at a distal site (Masse and Gottesman, 2002). The function of the *Salmonella* RyhB sRNAs was first described in two separate reports published in 2008 (Ellermeier and Slauch, 2008; Padalon-Brauch et al., 2008). The first of these reports demonstrated the effects of these sRNAs on the expression of *sodB*, encoding superoxide dismutase, and coined them the Rfr sRNAs (*R*NA for *F*ur *r*esponse) (Ellermeier and Slauch, 2008). The RfrA sRNA is most homologous to *E. coli* RyhB, sharing 82% sequence identity, and is encoded in the same genetic context as *E. coli* RyhB (Ellermeier and Slauch, 2008; Padalon-Brauch et al., 2008). RfrB, which is encoded on a *S. enterica* specific genetic island at a distal site on the chromosome, shares 53% sequence identity with RfrA, with the majority of sequence conservation spanning nucleotides 37 through 69. Shortly after the first report of these sRNAs, Padalon-Brauch et al. demonstrated iron regulation of both the RfrA and RfrB sRNAs and coined a separate name for the RfrB sRNA—IsrE (*i*sland-encoded *sR*NA) (Padalon-Brauch et al., 2008). For the purposes of this review, we will solely use the names RfrA and RfrB.

Similar to *E. coli*, the production of both RfrA and RfrB sRNAs is repressed by iron via the Fur protein (Ellermeier and Slauch, 2008; Padalon-Brauch et al., 2008; Kröger et al., 2013). RfrA and RfrB are also both induced by OxyR in response to oxidative stress, and as such play a role in mediating *Salmonella's* response to oxidative stress (Calderon et al., 2014a). Recently published studies further indicate that the Rfr sRNAs also mediate the response to nitrosative stress (Calderon et al., 2014b). In contrast to Fur and OxyR, the stationary phase sigma factor RpoS appears to play a more dominant role in promoting the production of RfrB than does iron limitation, indicating differential regulation of these sibling sRNAs (Padalon-Brauch et al., 2008). Production of both Rfr sRNAs is induced during infections of macrophages (Padalon-Brauch et al., 2008) and fibroblasts (Ortega et al., 2012). Thus, the production of at least one Rfr sRNA in *S. enterica* serovar Typhi is required for optimal growth inside macrophages, and either of the Rfr sRNAs can support this function (Leclerc et al., 2013). Moreover, the Rfr sRNAs of *S. enterica* serovar Typhimurium serve redundant functions in attenuating bacterial growth upon invasion of fibroblasts, a cell type in which growth is not advantageous to this organism (Ortega et al., 2012). While these studies suggest a role for the *Salmonella* Rfr sRNAs in pathogenesis, it is not yet clear if these functions are due to maintenance of iron homeostasis or regulation of virulence genes that are unique from those regulated by *E. coli* RyhB.

The studies discussed above indicate that the roles of the *Salmonella* Rfr sRNAs in pathogenesis are largely overlapping. However, some differences in the regulatory targets of the Rfr sRNAs have been noted. For instance, the RfrB sRNA affects the expression of a *Salmonella*-specific gene, STM1273, which is encoded on the strand opposite of the *rfrB* gene (Ortega et al., 2012). Specifically, the 5' end of STM1273 is antisense to the 5' end of RfrB, allowing for extensive complementarity with the sRNA. Due to the sequence dissimilarity of RfrA and RfrB in the 5' end of these sRNAs, RfrA shares only limited complementarity with STM1273, a feature that results in inefficient regulation of STM1273 by RfrA (Ortega et al., 2012; Kim and Kwon, 2013a). Kim and coworkers have additionally identified several motility genes (*flgJ, cheY*, and *fliF*), which are regulated by RfrB, but not RfrA, while other gene targets (*safA, acnB*) appear to be more strongly affected by the RfrA sRNA (Kim and Kwon, 2013b). The potential mechanisms responsible for the observed differential regulation by these two sRNAs have not yet been addressed. However, it appears that the sequence divergence in the 5' ends of the Rfr sRNAs may contribute to their ability to regulate the expression of distinct target mRNAs.

### *Yersinia* RyhB

The original *E. coli* RyhB study from Masse and Gottesman also noted that *Yersinia* species produce two RyhB sRNAs, and a subsequent study demonstrated that these sRNAs, designated RyhB1 and RyhB2, are indeed produced by *Yersinia* (Koo et al., 2011). Similarly to *Salmonella, Yersinia* RyhB1 is encoded within the same genetic context as *E. coli* RyhB, while RyhB2 is encoded at a distal site on the chromosome (Masse and Gottesman, 2002). RyhB1 and RyhB2 share an overall nucleotide identity of 69%, although the region predicted to mediate most target gene regulation is highly conserved between these two sRNA molecules. Additionally, while RyhB1 and RyhB2 share only 66 and 72% identity with *E. coli* RyhB, respectively, the conserved core region is nearly identical in each of these sRNA molecules (Deng et al., 2012).

Similarly to the *Salmonella* Rfr sRNAs, production of RyhB1 and RyhB2 is induced when *Yersinia pestis* is growing within the lungs of infected mice, although these sRNAs are not necessary for infection (Deng et al., 2012). To date, no regulatory targets of the *Y. pestis* RyhB1 or RyhB2 have been identified, so the question of whether these molecules have redundant or unique functions remains to be answered. However, sequence variation in the 5' region of the RyhB sRNAs may allow for differences in activity and/or stability. Indeed, the stability of RyhB1, but not that of RyhB2, is influenced by Hfq (Deng et al., 2012). Additionally, RyhB1 stability is more sensitive to perturbations in RNA degradosome factors than that of RyhB2 (Deng et al., 2014). It will be interesting to see what future investigations reveal about the significance of the sequence differences between RyhB1 and RyhB2, as well as how each of these two sRNAs influence the physiology and virulence of *Y. pestis*.

### *Pseudomonas* PrrF

The *Pseudomonas R*NAs responsive to iron (*F*e), or PrrFs, were described 2 years after the initial report of *E. coli* RyhB (Wilderman et al., 2004). The PrrF sRNAs of Pseudomonas species share no sequence homology with *E. coli* RyhB. Thus, authors of the initial report identified the PrrF sRNAs by searching the intergenic regions sequences of the *Pseudomonas aeruginosa* chromosome for a consensus Fur binding site and predicted Rho-independent terminator. This search revealed two sRNAs, PrrF1 and PrrF2, the production of which are induced by iron limitation and that function to block the expression of numerous iron-containing proteins. Only one report has been published describing the PrrF sRNAs produced by another Pseudomonad (Becerra et al., 2014), yet it is expected that the PrrF sRNAs universally mediate iron homeostasis in Pseudomonas species.

Remarkably, nearly all sequenced *Pseudomonas* species encode for sibling PrrF sRNA molecules. In *P. aeruginosa*, the PrrF1 and PrrF2 sRNAs are 95% identical to one another, and the function of these sibling sRNAs appears to be largely redundant: individual deletion of either *prrF* gene has little effect on expression of target genes or known phenotypes of the Δ*prrF1* Δ*prrF2* double mutant (Wilderman et al., 2004; Oglesby et al., 2008). In contrast to *P. aeruginosa*, the sequences of the sibling PrrF sRNAs of other Pseudomonads are more divergent, suggesting these sRNAs may be capable of regulating distinct targets. Additional studies into the function of the PrrF sRNAs in other Pseudomonads are needed to establish whether or not these sibling RNAs perform redundant functions in these species.

Curiously, the PrrF1 and PrrF2 sRNAs of *P. aeruginosa*, one sequenced strain of *Pseudomonas denitrificans*, and one sequenced strain of *Pseudomonas mendocina* are encoded in tandem, while all other sequenced Pseudomonas strains encoded for these sibling sRNAs at distal sites (Winsor et al., 2011). In *P. aeruginosa*, the tandem arrangement of *prrF1* and *prrF2* has been shown to allow for the expression of a third heme-regulated sRNA named PrrH (Oglesby-Sherrouse and Vasil, 2010). Transcription of PrrH in *P. aeruginosa* initiates at the 5' end of *prrF1*, proceeds through the *prrF1-prrF2* intergenic sequence (95 nt), and terminates at the 3' end of *prrF2* (Oglesby-Sherrouse and Vasil, 2010). Thus, expression of *prrH* is dependent on read-through transcription at the *prrF1* Rho-independent, or intrinsic, terminator. However, the mechanism by which heme regulates expression of PrrH remains unclear. Moreover, it is not known if *P. denitrificans* or *P. mendocina*, which also encode the PrrF1 and PrrF2 sRNAs in tandem, are capable of producing the PrrH sRNA.

Most studies of the PrrF1 and PrrF2 sRNAs of *P. aeruginosa* indicate the function of these sibling sRNAs is redundant. However, the PrrH sRNA includes sequence that is derived from the *prrF1-prrF2* intergenic region and thus is unique from the PrrF sRNAs, suggesting this sRNA may affect a distinct regulon. Evidence was previously provided that this unique region within PrrH may mediate regulation of *nirL*, encoding a regulator of heme *d*_1_ biosynthesis for anaerobic denitrification (Kawasaki et al., 1997). Thus, tandem arrangement of the sibling PrrF sRNAs appears to impart unique heme regulatory activities to *P. aeruginosa*. This hypothesis is particularly exciting in light of the fact that *P. aeruginosa* is the only human pathogen amongst the Pseudomonads, and that heme is an abundant source of iron during infection, suggesting a potential role for this sRNA in regulation of virulence-related genes. While more studies are needed to clarify the significance of the PrrH sRNA in *P. aeruginosa* physiology and virulence, this scenario underlines the potentially important role that chromosomal arrangement plays in the functioning of sibling sRNAs.

It is clear from the above referenced studies that many of the sibling RyhB orthologs and analogs characterized so far play redundant roles in mediating iron homeostasis. However, these studies have also revealed that the acquisition of a sibling iron-responsive sRNA can confer new regulatory activities through a variety of mechanisms. Particularly intriguing is how maintenance of these sibling sRNAs may be driven by the physiology of the species in which they are encoded. Iron strongly affects the virulence of the pathogenic species discussed above, and iron depletion is a well-conserved strategy of bacterial species to induce expression of virulence properties. Thus, expression of the sibling RyhB and RyhB-like sRNAs in iron-depleted environments may provide a competitive advantage to these pathogens during infection.

## OmrA and OmrB

The sRNAs now known as OmrA and OmrB were independently identified in *E. coli* by several of genome-wide screens including those based on conservation among species, the presence of “orphan” promoters and terminators, shot-gun cloning, and the ability of each molecule to interact with Hfq (Argaman et al., 2001; Wassarman et al., 2001; Vogel et al., 2003; Zhang et al., 2003). Encoded in tandem on the *E. coli* chromosome, OmrA and OmrB are 88 nt and 82 nt in length respectively, and share nearly identical nucleic acid sequences within the first 21 and last 35 nucleotides of each molecule (Wassarman et al., 2001; Guillier and Gottesman, 2006). The notable pattern of nucleic acid identity between OmrA and OmrB lead to the initial suggestion that these sibling sRNA molecules may function to regulate the expression of both shared and unique targets via their conserved and divergent sequences respectively (Guillier and Gottesman, 2006). *omrA* and *omrB* are well conserved amongst enterobacteriaceae, and in most cases are arranged in tandem on the bacterial chromosome. However, in at least one strain of *E. coli, omrA* and *omrB* are separated on the chromosome by an insertion element, and in the case of *Y. pestis* and *Erwinia carotovora* only one Omr sRNA is produced. The significance of the variability in number of Omr sRNAs and the arrangement of the corresponding genes to the physiology of enterobacteriaceae has yet to be revealed.

Initial experimental analyses demonstrated that while *E. coli* produces both OmrA and OmrB, their production varies uniquely in response to growth phase (Argaman et al., 2001; Wassarman et al., 2001; Zhang et al., 2003). Specifically, when cultured under nutrient rich conditions, OmrA levels peak during the stationary phase of growth while equally high levels of OmrB are observed throughout the logarithmic and stationary phases. Consistent with the observed difference in expression pattern is the finding that nucleic acid sequences within the *omrA* and *omrB* promoter regions vary (Guillier and Gottesman, 2006). Together these data suggest that the observed differences in growth-phase dependent OmrA and OmrB levels result from varying responsiveness of each promoter to changes in one or more environmental signal. Similarly, studies in *S. enterica* demonstrate that RybB and RybB-1, orthologs of *E. coli* OmrA and OmrB, display varied production patterns in response to a variety of environmental stressors including, but not limited to, cold shock and peroxide shock as well as changes in iron and oxygen availability (Kröger et al., 2013). In addition to differences in expression profiles, differences in stability of each *E. coli* sRNA is suggested by analysis during the late stationary phase of growth: OmrA accumulates in stationary phase, suggesting relative stability of this sRNA, while OmrB levels decrease, suggesting relative instability (Argaman et al., 2001; Vogel et al., 2003). Taken together, production, accumulation, and stability data highlight potentially important differences between OmrA and OmrB, and suggest that these highly homologous sibling sRNA molecules fulfill unique regulatory roles within the bacterial species in which they are produced.

Despite the differences highlighted above, subsequent characterization of OmrA and OmrB has revealed several similarities between theses sibling sRNA molecules. Firstly, production of both OmrA and OmrB is regulated by EnvZ and OmrR, the sensory kinase and response regulator composing a conserved two component regulatory system responsive to changes in osmolarity (Guillier and Gottesman, 2006). Secondly, the regulons of OmrA and OmrB, as identified to date, are identical and consists of genes encoding outer membrane proteins involved in iron acquisition (CirA, FecA, FepA) and protein degradation (OmpT), as well as *csgD* and *flhD*, encoding regulators of curli formation and motility, respectively (Guillier and Gottesman, 2006, 2008; Holmqvist et al., 2010; DeLay and Gottesman, 2012). Additionally, each Omr sRNA has been shown to repress expression of *ompR*, a gene encoding the response regulator controlling, among other genes, *omrA* and *omrB* expression (Guillier and Gottesman, 2008).

Investigations characterizing the molecular mechanism underlying OmrA- and OmrB-dependent regulation of each validated target have revealed the existence of several conserved characteristics including a dependence upon nucleic acid complementarity between the sRNA and the target transcript, the presence of a functional Hfq protein, and where investigated, the activity of RNaseE (Guillier and Gottesman, 2006, 2008; Holmqvist et al., 2010; DeLay and Gottesman, 2012). In at least one case, OmrA and OmrB have ben shown to regulate target gene expression by binding to, and modulating the structure of, a target mRNA molecule such that translation is blocked (Holmqvist et al., 2010).

All Omr-dependent regulation characterized to date has been shown to be mediated by conserved sequences located in the 5' region of each sRNAs. In some cases, investigations have been driven by the search for homology between the conserved region of the Omr sRNAs and putative target mRNA molecules using *in silico*-based approaches such as TargetRNA (Tjaden et al., 2006; Guillier and Gottesman, 2008). While these targeted approaches have proven fruitful in the identification of Omr-regulated gene targets, such a directed approach leaves open the distinct possibility that unique targets, presumably those regulated via sequences within the central variable region of each molecule, exist and are yet to be identified. Support for such a claim can be found in studies detailing the initial characterization of OmrA and OmrB, in which the authors presented a comprehensive list of genes for which a significant change in expression level was measured following increased production of each individual Omr sRNA molecule. Though not yet followed up on, the list of Omr-regulated genes contains some that appear to be influenced by OmrA but not by OmrB, and visa versa (Guillier and Gottesman, 2006). It will be exceptionally interesting to see if additional investigations into the regulation of these non-conserved targets reveal unique, non-redundant functions of OmrA and OmrB.

## AbcR sRNAs of the rhizobiales

Originally predicted in *Sinorhizobium meliloti* and designated as SmrC15 and SmrC16 (del Val et al., 2007), the AbcR1 and AbcR2 sibling sRNAs of the Rhizobiales order of the α-proteobacteria have been experimentally characterized in *Agrobacterium tumefaciens* (Wilms et al., 2011, 2012), *Brucella abortus* (Caswell et al., 2012), and *S. meliloti* (Torres-Quesada et al., 2013). The AbcR sRNA molecules are some of the most recent sRNAs to come to the stage of sibling sRNA research, and they provide a fascinating system with which to analyze the occurrence and importance of homologous sRNAs in closely related bacteria.

Wilms and colleagues first described the AbcR sRNAs of *A. tumefaciens*, where *abcR1* and *abcR2* are positioned in tandem on the *A. tumefaciens* chromosome (Wilms et al., 2011). Additionally, these sRNAs have similar predicted secondary structures and exhibit a high degree of identity (>65%) at the nucleotide level. In *Agrobacterium*, AbcR1, but not AbcR2, controls the expression of the ABC transporter-associated genes *atu0064* (*frcC*), *atu1879*, and *atu2422* by altering the stability of the mRNA messages, resulting in a negative impact on the expression of these genes, Atu2422 is a periplasmic-binding protein of an ABC transport system known to function in the import of γ-aminobutyric acid (GABA) by *Agrobacterium*, and the authors demonstrated that, in fact, AbcR1 controls GABA transport in *Agrobacterium*. More recently, AbcR1 from *A. tumefaciens* was shown to control the expression of multiple genes associated with ABC transport systems, and the authors demonstrated that two different regions within the AbcR1 sRNA sequence are responsible for controlling expression of these genes (Overlöper et al., 2014). While these studies provided the first functional characterizations of the AbcRs sRNAs in the α-proteobacteria, it is currently not known how, or if, the AbcRs contribute to the capacity of *A. tumefaciens* to infect plants.

The identification of AbcR1 and AbcR2 in *A. tumefaciens* precipitated the discovery of orthologous sRNAs in *B. abortus*, however there are striking differences between the AbcR sRNAs in these closely related bacteria (Caswell et al., 2012). In *Brucella*, AbcR1 and AbcR2 are >70% identical at the nucleotide level, but rather than being encoded in tandem, the *Brucella abcR1* and *abcR2* genes are located on different chromosomes (i.e., AbcR1 is encoded on chromosome 2, and AbcR2 in encoded on chromosome 1). Microarray and quantitative proteomic analyses revealed that the AbcR sRNAs in *B. abortus* control the expression of several genes encoding components of ABC transport systems, including *bab2_0612* and *bab1_1794*, which are the orthologs of the *A. tumefaciens* genes *atu1879* and *atu2422*, respectively, discussed in the previous paragraph. It was also determined that either AbcR1 or AbcR2 controls expression of some of these genes; however, the regulatory redundancy of the AbcR sRNAs for all of the genes in the AbcR regulon has not been confirmed. Binding of the *Brucella* AbcR sRNAs to their target mRNAs induces degradation of the target mRNAs, but the mechanism of mRNA degradation remains to be elucidated. Importantly, both AbcR sRNAs are required for the full virulence of *B. abortus*. Deletion of either *abcR1* or *abcR2* did not result in virulence defects, but deletion of both *abcR1* and *abcR2* led to significant attenuation of *B. abortus* in macrophages and mice, further highlighting the functional redundancy of the AbcRs in *Brucella*.

Most recently, the AbcR sRNAs of *S. meliloti* were described, and in *S. meliloti*, AbcR1 and AbcR2 exhibit >80% nucleotide identity (Torres-Quesada et al., 2013). Similar to the situation in *A. tumefaciens*, the *S. meliloti* AbcRs are encoded in tandem within the intergenic region flanked by genes encoding the ArsR and LysR transcriptional regulatory proteins. In *S. meliloti*, AbcR1, but not AbcR2, controls the expression of the periplasmic-binding protein LivK, which is the ortholog of Atu2422 of *A. tumefaciens* and BAB1_1794 of *B. abortus*. To date the mechanism by which the AbcR sRNAs regulate gene expression in *S. meliloti* is not known. The *S. meliloti* study also provided the first evidence that the AbcR sRNAs are differentially expressed in response to growth phase and various biologically relevant stimuli, as well as when the bacteria are inside the plant host. AbcR1, but not AbcR2, is produced during exponential growth, while AbcR2, but not AbcR1, is produced during stationary phase. Moreover, there is an inverse relationship between *abcR1* and *abcR2* expression during endosymbiosis with alfalfa roots, as AbcR1 was highly abundant, while AbcR2 is minimally detected, during symbiosis. The authors also demonstrated that a *S. meliloti abcR1* mutant strain exhibits a slight growth defect during laboratory culture; however, AbcR1 and AbcR2 are not required for *S. meliloti*-mediated nodulation of alfalfa roots.

*Agrobacterium, Brucella* and *Sinorhizobium* are close phylogenetic relatives, but they each have a very unique biology underscored by the different eukaryotic host that each bacterium interacts with, as well as the distinctive outcomes of those interactions. Nonetheless, these bacteria have maintained the AbcR sRNA system as a means of regulating the expression of several ABC transport systems. Very few of the AbcR-controlled ABC transport systems have been functionally characterized in terms of the substrates they transport, and it remains to be determined what the global contribution of AbcR-mediated regulation is to the physiological requirements of these bacteria. Additionally, it is not currently clear why the AbcR sRNAs in *Brucella* seem to exhibit regulatory redundancy while only AbcR1 appears to be functional in *Agrobacterium* and *Sinorhizobium*; on-going and future research assessing the molecular mechanisms of gene regulation by the AbcR sRNAs is expected to help answer these questions.

## Multiplicitous Qrr sRNAs of Vibrio spp

*Vibrio* species use chemical signals, termed autoinducers, to mediate cell-to-cell communication, a process referred to as quorum sensing (Bassler, 1999). For vibrios that are marine symbionts, quorum sensing controls bioluminescence, an important aspect of their symbiotic relationship with marine invertebrates. In the case of the human pathogens *Vibrio cholerae* and *Vibrio vulnificus*, these so-called quorum-sensing systems also function to regulate virulence gene expression. In 2004, Bassler and colleagues found that multiple, highly homologous sRNAs function to regulate the production of quorum sensing molecules in both *V. cholerae* and *Vibrio harveyi* (Lenz et al., 2004). These sibling sRNAs were named Qrr, for *q*uorum *r*egulatory *R*NAs, and were found to be encoded at various regions of the *V. cholerae* and *V. harveyi* genomes (Lenz et al., 2004; Tu and Bassler, 2007). The number of Qrr sRNAs encoded by each vibrio varies dramatically, with some species encoding 4 or 5 Qrr sRNAs (*V. vulnificus, V. cholerae, Vibrio parahaemolyticus*, and *V. harveyi*), while other species encode only one Qrr sRNA molecule (Tu and Bassler, 2007; Miyashiro et al., 2010; Shao and Bassler, 2012). At this time, it appears that all sequenced pathogenic Vibrio species encode multiple Qrr sRNAs (Bardill and Hammer, 2012).

When *Vibrio* are at low cell density, the two-component response regulator LuxO is phosphorylated and activates expression of the genes encoding the Qrr sRNAs (Lenz et al., 2004). Two of the first identified targets of the Qrr sRNAs were *hapR* in *V. cholerae* and *luxR* in *V. harveyi*, each encoding the major quorum sensing transcription factor in these species. Subsequent studies have also shown that several negative feedback loops function in each of these species to maintain the total levels of Qrr sRNA. One of these loops involves activation of *qrr* gene expression by HapR (*V. cholerae*) or LuxR (*V. harveyi*), which indirectly tempers HapR/LuxR expression (Svenningsen et al., 2008; Tu et al., 2008). The Qrr sRNAs of *V. cholerae* and *V. harveyi* also pair with and reduce expression of the *luxO* mRNA (Tu et al., 2008; Svenningsen et al., 2009). Combined, these feedback loops allow rapid attenuation of quorum sensing activities when cells are returned to low densities.

The Qrr sRNAs all contain a faithfully conserved 32-nucleotide core that is predicted to interact with many of the already-identified target mRNA molecules. By most accounts, regulation by the Qrr sRNAs of *V. cholerae* is thought to be functionally redundant, in that deletion of all four *qrr* genes is necessary to eliminate negative regulation of *hapR* (Lenz et al., 2004). This phenomenon is thought to be due to the negative feedback loops controlling *qrr* expression, as discussed above (Svenningsen et al., 2008, 2009). This is not the case in *V. harveyi*, in which regulation by the individual Qrr sRNAs appear to be additive, as shown by sequential deletion of all but one of this species *qrr* genes (Tu and Bassler, 2007). Curiously, production of Qrr5 has not been detected in wild type *V. harveyi* (Tu and Bassler, 2007), although induced expression of *qrr5* results in phenotypes similar to overproduction of the Qrr2-4 sRNAs (Tu and Bassler, 2007; Shao et al., 2013). At present, the function of the Qrr5 sRNA in regulation of *V. harveyi* quorum sensing remains unclear.

In spite of their largely redundant functions, a few studies have demonstrated that individual Qrr sRNAs differ in their ability to regulate distinct target mRNAs. The Qrr sRNAs of *V. harveyi* and *V. cholerae* activate expression of *aphA*, encoding a master regulator of genes important at low cell density. Most of the Qrr sRNAs possess two adjacent regions that pair with the *aphA* mRNA and mediate the observed regulation. However, the Qrr1 sRNAs from both *V. cholerae* and *V. harveyi* lack one of the regions with homology to *aphA* and, as a result, exert a more modest effect on *aphA* expression than their sibling sRNA molecules (Shao and Bassler, 2012). Additionally, two Qrr-repressed genes in *V. harveyi*, vibhar_00505 and vibhar_05691, are less well repressed by Qrr1 as compared to Qrr2-5 (Shao et al., 2013). Reduced regulation of these genes by Qrr1 is likely due to interaction with the 5' region of the Qrr2-5 sRNAs, which is lacking from the Qrr1 sRNA, allowing for differential regulation of these target mRNAs by the Qrr sRNAs. Thus, differences in both sequence and regulation of the Qrr sRNA molecules allow for both redundant and non-redundant functions in controlling the physiology and virulence of *Vibrio* species.

## csRNAs in *Streptococcus* species

The CiaRH two-component systems of *Streptococcus* species are global regulators of antibiotic resistance, competence, biofilm formation, virulence and several other physiological processes (Sebert et al., 2005; Halfmann et al., 2011; Li et al., 2011; Mazda et al., 2012). Some of the most highly regulated genes in the Cia regulon of different *Streptococcus* species encode regulatory RNAs, named csRNA (for *C*ia-dependent *sRNA*) (Halfmann et al., 2007; Marx et al., 2010) The csRNAs were the first identified in 2007 as part of an effort to define the CiaR regulons in *Streptococcus pneumoniae* (Halfmann et al., 2007). *S. pneumoniae* encodes 5 csRNAs (csRNA1-5), which share a significant amount of homolog. The csRNA5 molecule is the most divergent amongst these sRNA, due to an insertion of more than 50 nucleotides as compared to its siblings (Halfmann et al., 2007). Sequence homology amongst the csRNAs encoded by other *Streptococcus* species varies, yet all of the csRNAs contain conserved regions of homology (Marx et al., 2010).

Since their discovery in *S. pneumonia*, csRNAs have been detected in several other Streptococcal species, including *Streptococcus pyogenes, Stretpcoccus mitis, Streptococcos oralis*, and *Streptococcus sanguinis* (Perez et al., 2009; Marx et al., 2010). Moreover, the genomes of all sequenced Streptococcal species, both non-pathogenic and pathogenic, carry genes for at least two csRNAs, with some species encoding for as many as six (Marx et al., 2010). The genetic context of the genes encoding the csRNAs is also quite distinct amongst different Streptococcal species (Marx et al., 2010). In many species, several of the csRNAs are found clustered near *ruvB*, encoding a DNA helicase (Marx et al., 2010). In *S. pneumonia*, two of the csRNAs in this cluster are encoded in tandem, while the other csRNAs are encoded at distal sites (Halfmann et al., 2007). These reports outline the diversity in both sequence and localization of the sibling csRNAs in different Streptococcal species.

Phenotypic analysis of csRNA mutants in *S. pneumoniae* indicates that these sRNAs are capable of exerting effects on multiple cellular processes, including autolysis, competence, virulence, and β-lactam resistance (Halfmann et al., 2007; Tsui et al., 2010; Mann et al., 2012; Schnorpfeil et al., 2013). A recent report also demonstrated that the csRNAs could exert post-transcriptional control over several genes, and identified regions of significant complementarity between the csRNAs and these targeted mRNAs (Schnorpfeil et al., 2013). Results from this study also indicated that the csRNAs had redundant effects on these genes, raising questions about how some csRNAs affect certain phenotypes more strongly than others. Based on these studies in *S. pneumoniae*, it will also be interesting to determine if the csRNAs of other Streptococcal species also allow for non-redundant regulatory functions.

## GlmY and GlmZ

GlmY and GlmZ are highly homologous sibling sRNA molecules that, following initial identification in large-scale screens of the *E. coli* genome, were independently characterized and shown to influence the expression of *glmS*, a gene encoding the glucosamine 6-phosphate synthase enzyme (Argaman et al., 2001; Wassarman et al., 2001; Kalamorz et al., 2007; Urban et al., 2007). The enzymatic product of GlmS, glucosamine-6-phosphate (GlcN-6-P), is an essential component of the Gram-negative cell wall, making GlmS critical for survival of these organisms when external sources of the amino sugar are limiting. Interestingly, neither GlmY nor GlmZ influence the expression of *glmU*, an essential gene located immediately upstream of *glmS* on the same polycistronic *glmUS* transcript (Kalamorz et al., 2007; Urban et al., 2007). As such, GlmY and GlmZ became the first bacterial sRNA molecules shown to mediate dis-coordinate regulation of genes on a single polycistronic transcript in which the expression of only the down-stream cistron is activated via a post-transcriptional regulatory mechanism.

GlmY and GlmZ share extensive nucleic acid identity with each other, and initial studies demonstrated that increased production of either sRNA in *E. coli* results in increased expression of *glmS* (Kalamorz et al., 2007; Urban et al., 2007). While initial investigations revealed apparent redundancy in activity, subsequent studies have clearly demonstrated that *E. coli* GlmY and GlmZ are differentially produced and that each plays a critical and unique role in a hierarchical feedback loop that activates *glmS* expression in response to the intracellular concentrations of GlcN-6-P (Reichenbach et al., 2008; Urban and Vogel, 2008; Göpel et al., 2011). Once produced, GlmY functions as a molecular decoy that titrates detrimental processing machinery away from GlmZ. This GlmY-dependent protection of GlmZ facilitates an increase in the level of the latter sRNA that, in turn, directly activates the expression of *glmS* (Kalamorz et al., 2007; Urban et al., 2007; Reichenbach et al., 2008, 2009; Urban and Vogel, 2008; Göpel et al., 2013). In conjunction with Hfq, GlmZ interacts directly with the *glmS* transcript and promotes translation of the gene by both stabilizing the target message, and preventing the formation of an inhibitor structure that otherwise occludes the Shine-Delgarno sequence (Kalamorz et al., 2007; Urban and Vogel, 2008; Salim et al., 2012). Key to its effectiveness as an indirect regulator of GlmZ stability, levels of GlmY within the bacterial cell are dynamic. Both polyadenylation by PAP-1 and PNPase function to destabilize GlmY, and by doing so reset the responsiveness of this intricate regulatory cascade (Kalamorz et al., 2007; Andrade et al., 2012).

Until recently, *glmS* was the only recognized target of the GlmY and GlmZ regulatory cascade. It has now been demonstrated that in Enterohemorrhagic *E. coli* GlmY and GlmZ influence the expression of *espFu*, as well as several genes within the *l*ocus of *e*nterocyte *e*ffacement (LEE) pathogenicity island that encodes virulence factors required for attachment to mammalian cells (Gruber and Sperandio, 2014). Interestingly, the molecular mechanisms underlying Glm-dependent regulation of *espFu* and LEE genes are unique from each other, and also distinct from that underlying the regulation of *glmS* by these sRNA molecules (Gruber and Sperandio, 2014). In the case of *espFu*, both GlmY and GlmZ act to direct a cleavage event that promotes translation from the transcript. For genes within the LEE region, GlmY and GlmZ were found to mediate regulation by destabilizing the target transcripts. These recent findings regarding GlmY- and GlmZ-dependent regulation raise the possibility that the full impact of these conserved sibling sRNA molecules on bacterial physiology and virulence has yet to be revealed.

## 6S RNA

The subject of several recent reviews, 6S RNA is an exceptionally well-conserved sRNA that, despite controlling unique regulons within different species, invariably facilitates bacterial survival in a wide range of unique environments (Cavanagh and Wassarman, 2014; Steuten et al., 2014). Although discovered in the late 1960s, it would be 40 years before the regulatory function of *E. coli* 6S RNA would be elucidated (Hindley, 1967; Brownlee, 1971; Wassarman and Storz, 2000). In agreement with earlier studies indicating that 6S RNA is a member of a large ribonucleoprotein complex, initial studies into the function of this sRNA revealed that it specifically associates with, and alters the function of, RNA polymerase (Lee et al., 1978; Wassarman and Storz, 2000). It was more recently determined that activity of 6S RNA is strictly dependent upon proper assumption of a structure mimicing that of DNA within an open promoter complex; a largely double stranded molecule with a central single stranded bulge (Barrick et al., 2005; Trotochaud and Wassarman, 2005). Taken together, these studies demonstrate that by mimicking an open promoter complex, 6S RNA titrates RNA polymerase away from target promoters, and by doing so, modulates the expression of specific genes.

Specificity of 6S RNA-mediated regulation is due in part to conserved features within the regulated genes, and in part to the fact that the sRNA accumulates to high levels within *E. coli* as these cells reach the stationary phase of growth (Wassarman and Storz, 2000; Cavanagh et al., 2008). The relative abundance throughout the growth curve, together with its ability to sequester RNA polymerase, explains the finding that the 6S RNA regulon is composed of genes expressed during the stationary phase of growth (Trotochaud and Wassarman, 2004; Cavanagh et al., 2008). As such, it is not surprising that a functional 6S RNA has been correlated with fitness, facilitating growth of *E. coli* under the nutrient poor conditions encountered during entry into, and growth within stationary phase (Trotochaud and Wassarman, 2004). Beyond simply a molecular sponge to titrate RNA polymerase from target promoters throughout the stationary phase of growth, *E. coli* 6S RNA also functions as a template for the *de novo* synthesis of short product RNA (pRNA) molecules by the bound enzyme complex (Wassarman and Saecker, 2006; Gildehaus et al., 2007). pRNA synthesis occurs during outgrowth from stationary phase and is thought to destabilize the interaction between 6S RNA and RNA polymerase, releasing the enzyme and functionally resetting the regulatory circuit (Wassarman and Saecker, 2006; Gildehaus et al., 2007; Cavanagh et al., 2012).

While initially identified and characterized in *E. coli*, subsequent *in silico* and biochemical studies have demonstrated that 6S RNA is exceptionally well conserved in a wide variety of bacterial species (Barrick et al., 2005; Trotochaud and Wassarman, 2005). While the structure and basic mechanism of action appear to be conserved, the production profile, regulon identity and physiological impact of 6S RNA varies (Cavanagh and Wassarman, 2014). Cellular processes that are influenced by 6S RNA include, but are not limited to, sporulation, intracellular survival and various stress responses (Faucher et al., 2010; Peeters et al., 2010; Cavanagh and Wassarman, 2013; Venkataramanan et al., 2013). Interestingly, several bacterial species, including but not limited to *Bacillus subtilis* and *Legionella pneumophila*, produce sibling 6S RNA molecules (Ando et al., 2002; Barrick et al., 2005; Trotochaud and Wassarman, 2005; Faucher et al., 2010; Faucher and Shuman, 2011; Weissenmayer et al., 2011). The most well characterized set of sibling 6S RNA molecules are 6S-1 and 6S-2 of *B. subtilis*. Like other 6S RNAs characterized to date, *B. subtilis* 6S1-RNA and 6S2-RNA share a conserved structure, bind with high affinity to RNA polymerase, and function as templates for the synthesis of pRNA, albeit at different efficiencies (Barrick et al., 2005; Trotochaud and Wassarman, 2005; Beckmann et al., 2011; Cavanagh et al., 2012; Cabrera-Ostertag et al., 2013; Burenina et al., 2014). In additions to findings that demonstrate unique expression profiles, preliminary reports suggest that the 6S-1 and 6S-2 RNAs have unique regulons, and thus unique functions in *B. subtilis* (Ando et al., 2002; Barrick et al., 2005; Trotochaud and Wassarman, 2005; Beckmann et al., 2011; Cavanagh et al., 2012).

Initial studies aimed at identifying sRNAs involved in pathogenesis of *L. pneumophila* implicated 6S RNA as a regulator of virulence gene expression (Faucher et al., 2010). Specifically, 6S RNA was found to regulate the expression of genes encoding factors involved in a variety of essential processes including stress response and nutrient acquisition as well as genes encoding effectors of the type IVB secretion system. While the loss of 6S RNA had no impact on the ability of *L. pneumophila* to grow when cultured in rich media, the mutant strain displayed decreased intracellular multiplication within both protozoan and mammalian cells as compared to growth of the wild-type strain. These initial studies clearly demonstrated 6S RNA as a *L. pneumophila* virulence factor. Interestingly, transcriptome analyses later revealed that like *B. subtilis, L. pneumophila* produces two 6S-RNA molecules; 6S1 and 6S2 (Weissenmayer et al., 2011). Though little is known about the relative activity of 6S1 and 6S2, expression analysis suggests that the sibling sRNAs are uniquely regulated in response to growth phase; a finding that suggests that each plays a unique role in regulating *L. pneumophila* gene expression (Weissenmayer et al., 2011).

In cases where sibling 6S RNA molecules have been identified and characterized, it appears that, despite the presence of universally conserved features, these riboregulators are differentially regulated and/or control the expression of non-redundant regulons. While much has been revealed, it is clear that more questions remain than have been answered regarding the potentially unique roles that sibling 6S sRNAs play in controlling the physiology of the species in which they are found.

## Csr/Rsm sRNAs

In addition to those that regulate via complementary base pairing, several bacterial species encode sibling sRNA molecules that regulate gene expression by binding to, and altering the function of, regulatory proteins. The best-studied examples of protein binding sRNAs are those that are members of the Csr (carbon storage regulator) and Csr-like family of sRNA regulators. These systems are composed of a post-transcriptional regulatory protein, which binds to and affects the stability of several target mRNAs. In 1997, Romeo and colleagues found that the CsrA regulatory protein of *E. coli* co-purified with a non-coding sRNA, named CsrB (Liu et al., 1997). Subsequent studies have established that the regulatory activity of CsrA is modulated by CsrB, as well as a sibling sRNA, named CsrC. To date, Csr systems have been described in numerous bacterial species, in some cases named Rsm (for *r*epressor of *s*tationary-phase *m*etabolites), and several reviews have been published that thoroughly describe how these systems affect the physiology and virulence of the organisms in which they are produced (Lucchetti-Miganeh et al., 2008; Heroven et al., 2012; Romeo et al., 2013). Here we will focus on the sibling nature of these sRNAs in several bacterial species, and how their distinct mechanism of regulation may allow for characteristics that are unique from other sibling sRNAs described in this review.

The Csr/Rsm sRNAs have been identified in multiple Gram-negative bacteria, including several members of Enterobacteriaceae (Liu et al., 1997; Lenz et al., 2005; Kulkarni et al., 2006), *Pseudomonas* (Kay et al., 2005, 2006), and *Legionella* (Sahr et al., 2009) species. The number of known Csr/Rsm sRNAs encoded by individual species varies from one (*E. caratovora*, Liu et al., 1998) to three (*V. cholera*, Lenz et al., 2005, *and Pseudomonas fluorescens*, Kay et al., 2005), although most species studied have been shown to encode for at least two of these sRNAs. Of note, the sequence divergence amongst the Csr/Rsm sRNAs encoded by a single bacterium is greater than other sibling sRNAs discussed in this review, which is likely attributed to the fact that these sRNAs do not rely on base pairing for regulation. Instead, the Csr/Rsm sRNAs take advantage of GGA motifs, located in the hairpin loops or unstructured regions of the sRNAs, which bind to and sequester the CsrA/RsmA RNA-binding proteins away from their regulatory target mRNA molecules.

What remains unclear is whether sequestration of CsrA/RsmA is the sole mechanism by which the Csr/Rsm sRNAs regulate gene expression. Are these sRNAs also capable of pairing with complementary regions of target mRNAs? There exists at least one example of an sRNA that can regulate gene expression by both protein titration and complementary base pairing with target mRNAs (Jørgensen et al., 2013). If this is also the case for the Csr/Rsm sRNAs, does their sequence divergence allow them to regulate distinct regulons? Some evidence exists that suggests that certain Csr/Rsm-related phenotypes may be due to only one of the sibling sRNAs encoded by certain bacteria. For example, *P. aeruginosa* biofilm formation is dependent upon post-transcriptional down-regulation of the RsmZ sRNA, but not its sibling RsmY sRNA (Petrova and Sauer, 2010). Additionally, divergence in the promoter structures of the *P. aeruginosa rsmY* and *rsmZ* genes allows for differential regulation of the encoded sRNAs (Brencic et al., 2009; Bordi et al., 2010). Thus, while the Csr/Rsm RNAs are often considered to be functionally redundant, differential expression and target selection suggest possible non-redundant roles for these sibling RNAs.

## Discussion

### The elephant in the room: why have sibling sRNA systems evolved?

The questions that we often ask ourselves (and that we ask each other) when working with and studying sibling sRNAs are: How and why have these systems evolved and been maintained by numerous, phylogenetically unrelated bacteria? In truth, “how” and “why” they have evolved are very different questions. In regards to “how,” it is likely that these sRNAs are the result of gene duplication events. A recent study employing mathematical modeling of *E. coli* and *Shigella* genomes suggests that the sibling sRNAs OmrA and OmrB arose from gene duplication (Skippington and Ragan, 2012). Similarly, Gottesman and Storz propose that the PrrF sRNAs resulted from gene duplication, and the movement of the *prrF* genes within the genome was secondary to the duplication event (Gottesman and Storz, 2011). Thus, it is highly likely that sibling sRNAs in general came about as a result of duplication of a single sRNA-encoding gene, and in the cases where the two sRNAs are not found in tandem or in the same region of the genome, that the movement of one sibling sRNA to another locus was subsequent to the duplication event. While the likelihood is low, it is also possible that some sibling sRNAs evolved independently from separate lateral gene transfer events and not from a gene duplication event, but there is no compelling evidence for this scenario. Overall, we can presently only speculate about these events, and more work is needed to establish legitimate hypotheses as to the mechanism(s) of the evolution of sibling sRNAs.

**Figure 1:**
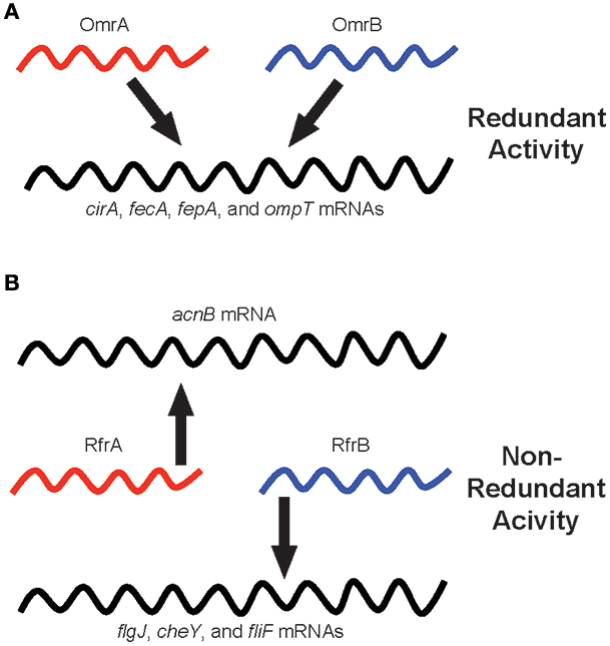
**Regulatory redundancy and non-redundancy of bacterial sibling sRNAs**. Individual sibling sRNAs are capable of controlling the expression of genes similarly to their sibling(s) (i.e., redundant regulation), but sibling sRNAs are also capable of regulating the expression of unique sets of genes as compared to their sibling(s) (i.e., non-redundant regulation). The schematic illustrates sibling sRNAs in red and blue, and their regulatory relationships to target mRNAs, depicted in black. **(A)** The OmrA and OmrB sibling sRNAs of *Escherichia coli*: A model for redundant sibling sRNAs. **(B)** The RfrA and RfrB sibling sRNAs of *Salmonella enterica*: a model for non-redundant sibling sRNAs.

Why have sibling sRNAs evolved? This is a more challenging and provocative question compared to the “how” of evolution, but there are a couple of viable hypotheses as to the reasons for acquiring and maintaining multiple copies of highly similar sRNA-encoding genes (Figure 1). First, in cases where sibling sRNAs exhibit regulatory redundancy, it is possible that having multiple copies ensures the downstream system controlled by the sRNAs is intact for essential processes. The OmrA and OmrB sibling sRNAs of *E. coli* are classic examples, as each of these sRNAs regulates the expression of iron-homeostasis genes, including *cirA, fecA, fepA*, and *ompT* (Figure 1A). Additionally, having multiple copies of functionally identical sRNAs may allow for differential regulation of expression of the sRNAs themselves. By regulating the expression of individual sibling sRNA genes with different, specific signals, the common target gene(s) controlled by the sRNAs can be appropriately expressed in response to numerous stimuli encountered by the bacterium.

The *S. enterica* sibling sRNAs, RfrA, and RfrB, illustrate non-redundant bacterial sRNAs, as only RfrA controls the expression of *acnB*, a gene encoding aconitase, while RfrB uniquely regulates *flgJ, cheY*, and *fliF*, which are involved in motility and chemotaxis (Figure 1B). In situations where the sibling sRNAs display different regulatory activities from one another, it is likely that the additional copy of the sRNA-encoding gene has been retained in the genome only to be altered for a function distinct from the “original sibling” sRNA. In these cases, the new version of the sRNA has been slightly changed by the accumulation of small alterations in the nucleotide sequence leading to different regulatory interactions, and, in turn, vastly different functional outcomes. The selective pressures driving these types of modifications to the regulatory and functional activities of sibling sRNAs are not clear. However, it is possible that rather than simply delete the duplicated, potentially unnecessarily redundant gene from the genome, the bacterium benefited from the increased regulatory diversity that resulted from the slightly altered version of the duplicated sRNA gene. Overall, whether sibling sRNAs are functionally redundant or non-redundant can be determined empirically, but it is hopeful that future studies will also shed light on the evolutionary reasons for generating and maintaining sibling sRNAs.

### Conflict of interest statement

The authors declare that the research was conducted in the absence of any commercial or financial relationships that could be construed as a potential conflict of interest.
